# Cardioprotective and Antioxidant Effects of Marine-Derived *Xestospongia testudinaria* in an Isoprenaline-Induced Rat Model of Heart Failure

**DOI:** 10.3390/ph19071030

**Published:** 2026-06-30

**Authors:** Rajasegar Anamalley, Iman Nabilah Abd Rahim, Siti Nurhannah Irdina Hasmar Honiz, Stephenie Tamil Many, Partiban Manoharan, Satirah Zainalabidin, Sasimalani Surgunnam, Nurzafirah Mazlan, Fikri Akmal Khodzori, Muhammad Dawood Shah, Rahayu Zulkapli

**Affiliations:** 1 Faculty of Health and Life Sciences, Management and Science University, Shah Alam 40100, Selangor, Malaysiasasimalani@msu.edu.my (S.S.); 2 Iman NaturePro, Bandar Indera Mahkota, Kuantan 25200, Pahang, Malaysia; 3 Centre of Toxicology and Health Risk Study (CORE), Universiti Kebangsaan Malaysia, Kuala Lumpur 50300, Federal Territory of Kuala Lumpur, Malaysia; satirah@ukm.edu.my; 4 Borneo Marine Research Institute, Universiti Malaysia Sabah, Kota Kinabalu 88400, Sabah, Malaysia; nurzafirah@ums.edu.my (N.M.); mfakmal@ums.edu.my (F.A.K.); dawoodshah@ums.edu.my (M.D.S.); 5 Faculty of Dentistry, Universiti Teknologi MARA, Sungai Buloh 47000, Selangor, Malaysia; rahayu88@uitm.edu.my; 6 Cardiovascular Advancement and Research Excellence Institute (CARE Institute), Universiti Teknologi MARA, Sungai Buloh 47000, Selangor, Malaysia

**Keywords:** *Xestospongia testudinaria*, heart failure, oxidative stress, cardioprotection, isoprenaline, KEAP1, NRF2, marine natural products, antioxidant activity

## Abstract

**Background:** Oxidative stress and maladaptive cardiac remodeling are key contributors to heart failure progression. *Xestospongia testudinaria*, a marine sponge rich in bioactive compounds, possesses antioxidant and lipid-modulating properties. This study investigated the cardioprotective and antioxidant effects of *Xestospongia testudinaria* methanolic extract (Xesto) in an isoprenaline-induced rat model of heart failure. **Methods:** Thirty-five healthy male Wistar rats weighing 200–250 g were used in this study. Heart failure was induced in rats via subcutaneous administration of isoprenaline (10 mg/kg/day) for 14 days. Rats were subsequently treated with Xesto (15 mg/kg/day, oral gavage), digoxin (10 mg/kg/day), or saline for an additional 14 days. Hemodynamic parameters, serum NT-proBNP, oxidative stress biomarkers, biochemical indices, hematological parameters, and histopathological changes were evaluated. Molecular docking was performed to assess the interaction of Xesto constituents with Kelch-like ECH-associated protein 1 (KEAP1). **Results:** Isoprenaline administration significantly increased blood pressure, NT-proBNP, malondialdehyde, hepatic enzymes, and urea levels, while reducing superoxide dismutase and catalase activities. Xesto treatment significantly improved hemodynamic parameters, restored antioxidant enzyme activities, reduced lipid peroxidation, and normalized biochemical and hematological alterations. Histological analysis demonstrated reduced cardiomyocyte hypertrophy and collagen deposition in Xesto-treated rats. Docking analysis showed favorable binding of trans-phytol within the KEAP1 Kelch domain, suggesting possible modulation of antioxidant regulatory pathways. **Conclusions:** *Xestospongia testudinaria* exhibited significant cardioprotective and antioxidant effects in isoprenaline-induced heart failure, potentially through enhancement of endogenous antioxidant defenses and attenuation of pathological cardiac remodeling. These findings support its potential as a marine-derived therapeutic candidate for oxidative stress-associated cardiovascular disorders.

## 1. Introduction

Cardiovascular diseases (CVDs) remain one of the leading causes of morbidity and mortality worldwide, encompassing a broad spectrum of cardiac dysfunctions, including heart failure (HF) [1,2]. HF is a progressive clinical syndrome characterized by the heart’s inability to pump blood effectively, resulting in reduced exercise tolerance, recurrent hospitalization, and diminished quality of life [3,4]. Despite advances in pharmacological and device-based therapies, the global burden of HF continues to increase, highlighting the need for novel and adjunctive therapeutic strategies that target underlying pathogenic mechanisms.

Among the multifactorial processes involved in HF progression, oxidative stress is recognized as a central driver of myocardial injury and adverse remodeling. Oxidative stress arises from an imbalance between reactive oxygen species (ROS) production and endogenous antioxidant defenses, leading to lipid peroxidation, mitochondrial dysfunction, inflammation, and cardiomyocyte apoptosis [5,6,7]. Under physiological conditions, antioxidant enzymes such as superoxide dismutase (SOD) and catalase (CAT) maintain redox homeostasis. However, sustained ROS overproduction in HF overwhelms these systems, contributing to ventricular hypertrophy, fibrosis, and impaired contractility. Increasing evidence indicates that modulation of oxidative signaling pathways, particularly the Kelch-like ECH-associated protein 1 (KEAP1)–nuclear factor erythroid 2-related factor 2 (NRF2) axis, represents a promising therapeutic target. KEAP1 functions as a cytoplasmic inhibitor of NRF2, promoting its ubiquitination and degradation. Disruption of the KEAP1–NRF2 interaction allows NRF2 nuclear translocation and transcriptional activation of antioxidant response genes, including SOD and CAT, thereby enhancing cytoprotection against oxidative stress.

Experimental animal models remain essential for investigating the pathophysiology of heart failure and evaluating potential therapeutic interventions [8,9]. The isoprenaline (ISO)-induced rat model is widely used because it reproduces several key features of human heart failure, including oxidative stress, myocardial hypertrophy, fibrosis, ventricular dysfunction, and elevated cardiac injury biomarkers [10]. These pathological changes closely resemble mechanisms involved in the progression of human cardiovascular disease, making this model suitable for assessing the cardioprotective and antioxidant effects of novel therapeutic candidates.

Digoxin, a cardiac glycoside derived from Digitalis lanata, remains in clinical use for selected HF patients, particularly when conventional therapies are insufficient [11,12]. Its positive inotropic effects improve cardiac output through Na^+^/K^+^-ATPase inhibition, while its atrioventricular nodal effects reduce heart rate [13]. However, the clinical utility of digoxin is limited by its narrow therapeutic window and risk of toxicity, including arrhythmias and sudden cardiac death [13,14]. These safety concerns underscore the need for alternative cardioprotective agents with broader therapeutic margins and additional antioxidative benefits.

Natural products have gained increasing attention as reservoirs of structurally diverse bioactive compounds capable of modulating oxidative and inflammatory pathways [15]. Marine organisms, particularly sea sponges of the genus Xestospongia, produce a wide array of secondary metabolites, including polyphenols, quinones, and terpenoids, with demonstrated antioxidant and cytoprotective activities [16]. Previous investigations have shown that compounds isolated from Xestospongia species exhibit lipid-lowering and antiatherosclerotic properties. For example, Fraction-7 from Xestospongia muta upregulated scavenger receptor class B type I (SR-BI), enhancing HDL-mediated cholesterol uptake [17], while ethanolic extracts reduced total cholesterol and triglycerides in hyperlipidemic models [18]. These findings align with broader evidence supporting marine-derived bioactives as modulators of oxidative stress, inflammation, and endothelial dysfunction [19,20].

Despite these promising observations, the cardioprotective effects of *Xestospongia testudinaria* in heart failure remain largely unexplored, and the molecular mechanisms underlying its antioxidant activity have not been systematically investigated. In particular, whether bioactive constituents of Xestospongia interact with redox-regulatory targets such as KEAP1 to influence NRF2-mediated antioxidant responses has not been examined.

Accordingly, the present study aims to evaluate the cardioprotective potential of the methanolic extract of *Xestospongia testudinaria* (Xesto) in an isoprenaline (ISO)-induced rat model of heart failure and to investigate its possible molecular mechanism. Hemodynamic, biochemical, antioxidant, and histopathological parameters were assessed to determine functional cardioprotection. To explore the underlying antioxidant mechanism, molecular docking was performed to examine potential interactions between major Xesto-derived compounds and KEAP1, a key regulator of the NRF2-mediated antioxidant response. The novelty of this study lies in integrating in vivo cardioprotection with in silico mechanistic interrogation of the KEAP1–NRF2 axis, providing initial evidence that Xesto may exert its effects through coordinated antioxidant modulation and potential interference with KEAP1-mediated NRF2 suppression.

## 2. Results

### 2.1. Molecular Docking Analysis

Docking analysis revealed differential binding affinities of the tested compounds toward KEAP1. The reference ligand digoxin demonstrated the strongest binding affinity with a binding energy of −10.8 kcal/mol. Among the Xesto-derived compounds, trans-phytol exhibited moderate binding (−6.3 kcal/mol), whereas nonanedioic acid showed lower binding affinity (−4.3 kcal/mol). A summary of binding energies and key interacting residues is presented in Table 1.

Digoxin formed multiple stabilizing interactions within the KEAP1 binding pocket. Conventional hydrogen bonds were observed with ARG415 and TYR525, while additional stabilizing contacts involved ASN382, ASN414, SER363, GLY364, GLY603, ALA556, TYR572, and GLN530. Hydrophobic and π-interactions were detected with TYR334 and surrounding residues within the Kelch domain. These extensive interactions likely contributed to its strong binding energy.

Trans-phytol demonstrated a moderate binding profile within the Kelch domain of KEAP1. A conventional hydrogen bond was observed with ARG415. Hydrophobic and alkyl interactions were detected with TYR334, TYR572, ALA556, and PHE577, along with van der Waals interactions involving ASN382, ASN414, SER363, GLY462, GLY509, SER508, SER555, GLN530, and TYR525. The predominance of hydrophobic contacts suggests that trans-phytol stabilizes within the binding cavity primarily through non-polar interactions.

Nonanedioic acid exhibited fewer stabilizing interactions compared to the other ligands. A hydrogen bond was observed with GLU493, along with van der Waals interactions involving ARG470, ASN469, VAL539, TYR473, TYR491, ARG494, and PRO492. Hydrophobic contact was also detected with LEU471. The limited interaction network likely accounts for its comparatively lower binding affinity.

**Figure 1:**
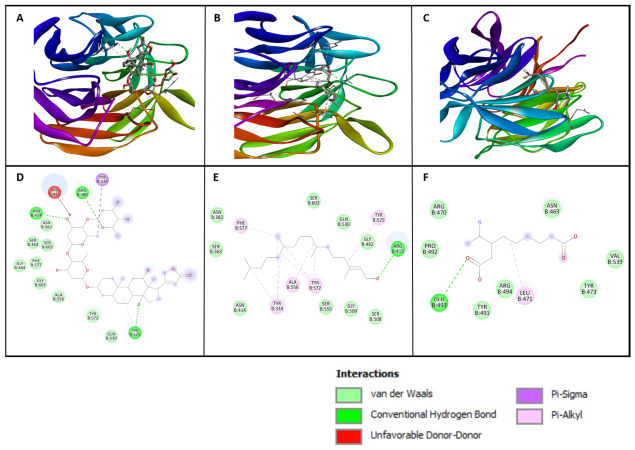
Molecular docking analysis of digoxin and Xesto-derived compounds with KEAP1. Top panels (**A**–**C**) show the three-dimensional binding poses of digoxin, trans-phytol, and nonanedioic acid within the Kelch domain of KEAP1. Bottom panels (**D**–**F**) present the corresponding two-dimensional interaction diagrams illustrating hydrogen bonds, van der Waals interactions, and hydrophobic contacts between each ligand and key amino acid residues. In the two-dimensional interaction diagrams, dashed lines represent ligand–protein interactions, whereas solid lines represent the chemical structure of the ligand. Notably, both digoxin and trans-phytol interacted with key residues within the Kelch domain, particularly ARG415 and surrounding aromatic residues implicated in NRF2 recognition. These findings suggest that trans-phytol may potentially interfere with the KEAP1–NRF2 interaction, thereby supporting the antioxidant and cytoprotective effects observed in vivo.

Three-dimensional binding poses and corresponding two-dimensional interaction maps are illustrated in Figure 1, showing the localization of each ligand within the Kelch domain and their respective hydrogen bonding and hydrophobic interactions.

### 2.2. Phytochemical Screening Analysis

The qualitative phytochemical screening of the methanolic extract of *Xestospongia testudinaria* indicated the presence of phenolics and tannins, saponins, triterpenoids, and steroids, while alkaloids, flavonoids, and cardiac glycosides were absent (Table 2).

### 2.3. Total Phenolic Content (TPC) and Total Flavonoid Content (TFC)

The total phenolic content of the methanolic extract of *Xestospongia testudinaria* at different concentrations is presented in Table 3.

**Table 3:** The total phenolic content of the methanolic extract of *Xestospongia testudinaria* at different concentrations.

Concentration (μg/mL)	TPC of Methanol Extract of *Xestospongia testudinaria* (mg GAE/100 g ± SEM)
25	1299 ± 5.94
50	1222 ± 6.43
75	3073 ± 6.80
100	4238 ± 10.52
125	5667 ± 9.09
150	6575 ± 8.81

The extract exhibited the highest TPC value of 6575 ± 8.81 mg GAE/100 g, showing a concentration-dependent increase. The total flavonoid content of the extract, shown in Table 4, ranged from 2.20 ± 0.04 to 14.46 ± 0.02 mg QE/100 g, which was comparatively lower than the TPC values.

**Table 4:** Total flavonoid content (TFC) of methanol extract of *Xestospongia testudinaria*.

Concentration (mg/mL)	TFC of Methanol Extract of *Xestospongia testudinaria* (mg QUE/100 g ± SEM)
0.1	2.20 ± 0.04
0.2	4.99 ± 0.04
0.4	6.80 ± 0.04
0.6	10.94 ± 0.02
0.8	14.46 ± 0.02

### 2.4. Bodyweight Analysis

**Figure 2:**
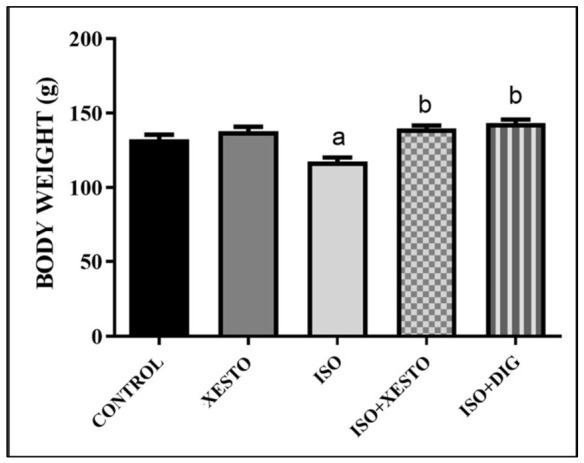
Effects of Xesto on body weight in each experimental group. Data are expressed as mean ± SEM (*n* = 7). Statistical significance is indicated as: a *p* < 0.05 vs. Control; b *p* < 0.05 vs. ISO. CONTROL: rats given normal saline. XESTO: rats given *Xestospongia testudinaria* extract (15 mg/kg/day). ISO: rats administered isoprenaline (10 mg/kg/day). ISO + XESTO: rats administered ISO followed by Xesto extract (15 mg/kg/day). ISO + DIG: rats administered ISO followed by digoxin (10 mg/kg/day).

The body weight of Wistar rats significantly increased after induction with ISO compared to the control group (*p* < 0.05). Treatment with *Xestospongia testudinaria* extract (ISO + Xesto) or digoxin (ISO + Dig) significantly restored body weight compared to the ISO group (*p* < 0.05). No significant difference in body weight was found between the control, control + Xesto, ISO + Xesto, and ISO + Dig groups (Figure 2).

### 2.5. Heart Weight Analysis

**Figure 3:**
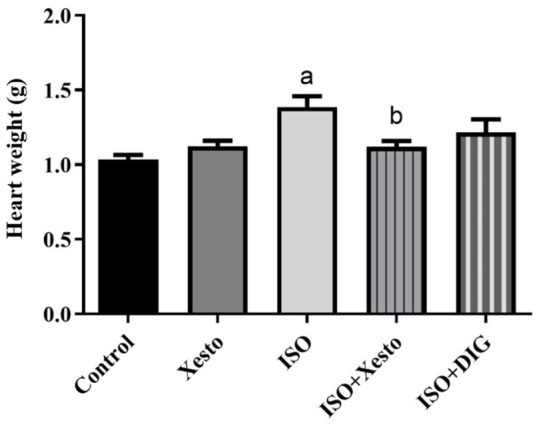
Effects of Xesto on heart weight of rats in each experimental group. Data are expressed as mean ± SEM (*n* = 7 per group). Statistical significance is indicated as: a *p* < 0.05 vs. Control; b *p* < 0.05 vs. ISO. Control: rats given normal saline. Xesto: rats given *Xestospongia testudinaria* extract (15 mg/kg/day). ISO: rats administered isoprenaline (10 mg/kg/day). ISO + Xesto: rats administered ISO followed by Xesto extract (15 mg/kg/day). ISO + DIG: rats administered ISO followed by digoxin (10 mg/kg/day).

The ISO-treated rats exhibited a marked increase in heart weight compared to the control group (*p* < 0.05), confirming the induction of cardiac hypertrophy (Figure 3). Administration of *Xestospongia testudinaria* extract (ISO + Xesto) or digoxin (ISO + Dig) significantly lowered heart weight relative to the ISO group (*p* < 0.05). There was no significant difference in heart weight among the control, Xesto-only, ISO + Xesto, and ISO + Dig groups.

### 2.6. Systolic and Diastolic Blood Pressure Analyses

**Figure 4:**
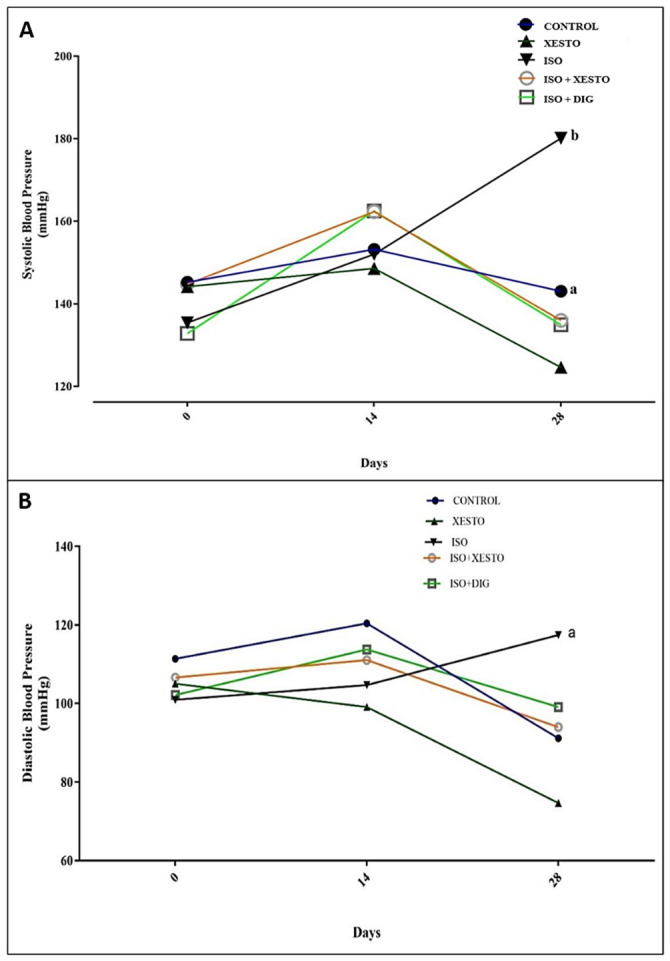
Effects of Xesto on blood pressure in rats from each experimental group measured at baseline (Day 0), Day 14, and Day 28. (**A**) Systolic blood pressure (SBP). (**B**) Diastolic blood pressure (DBP). Data are expressed as mean ± SEM (*n* = 7 per group). Statistical significance is indicated as: a *p* < 0.05 vs. Control; b *p* < 0.05 vs. ISO. CONTROL: rats given normal saline. XESTO: rats given *Xestospongia testudinaria* extract (15 mg/kg/day). ISO: rats administered isoprenaline (10 mg/kg/day). ISO + XESTO: rats administered ISO followed by *Xestospongia testudinaria* extract (15 mg/kg/day). ISO + DIG: rats administered ISO followed by digoxin (10 mg/kg/day).

A significant elevation in systolic blood pressure was observed in the ISO-treated group on day 28 compared to the control group (*p* < 0.05), confirming the development of hypertension. In contrast, the ISO + Xesto and ISO + Dig groups showed a significant reduction in SBP after day 14 and on day 28 compared to the ISO group (*p* < 0.05), indicating an improvement following treatment. No significant differences were observed between the control and Xesto-only groups throughout the study (Figure 4A). Diastolic blood pressure (DBP) followed a similar trend (Figure 4B). The ISO group demonstrated a progressive increase in DBP, with a significant rise on day 28 compared to the control group (*p* < 0.05). Both ISO + Xesto and ISO + Dig groups exhibited a marked reduction in DBP after day 14, maintaining levels closer to the control group.

### 2.7. Biochemical Analysis

Serum biochemical analysis revealed significant alterations in liver and renal function markers following isoprenaline (ISO) administration (Table 5). The ISO-treated group showed marked increases in aspartate aminotransferase (AST; 115.4 ± 2.96 U/L) and alanine aminotransferase (ALT; 89.43 ± 1.56 U/L) compared to the control group (*p* < 0.05), indicating hepatic stress. Similarly, serum urea levels were elevated (7.89 ± 0.22 mmol/L) relative to the control group (5.94 ± 0.16 mmol/L, *p* < 0.05).

Treatment with Xesto or digoxin significantly attenuated these elevations to varying degrees. The ISO + Xesto group showed reduced AST (105.7 ± 2.58 U/L), ALT (74.29 ± 1.60 U/L), and urea (7.10 ± 0.11 mmol/L) levels compared to the ISO group (*p* < 0.05). More pronounced reductions were observed in the ISO + Dig group (96.29 ± 1.57 U/L, 60.57 ± 1.29 U/L, and 6.26 ± 0.21 mmol/L, respectively). These findings suggest potential hepatoprotective and renoprotective effects of Xesto against ISO-induced toxicity.

### 2.8. Hematological Analysis

The haematological analysis revealed significant alterations following isoprenaline (ISO) administration (Table 6). Rats in the ISO group exhibited a marked decrease in haemoglobin concentration (11.88 ± 0.24 g/dL) compared to the control group (16.13 ± 0.50 g/dL, *p* < 0.05). Treatment with Xesto (13.48 ± 0.19 g/dL) or digoxin (15.10 ± 0.24 g/dL) significantly restored haemoglobin levels relative to the ISO group (*p* < 0.05).

Leukocyte and neutrophil counts were substantially elevated in the ISO group (15.42 ± 0.44 × 10^9^/L and 8.20 ± 0.15 × 10^9^/L, respectively) compared to the control (*p* < 0.05), suggesting an ISO-induced inflammatory response. Administration of Xesto markedly reduced both leukocyte (9.90 ± 0.30 × 10^9^/L) and neutrophil (6.12 ± 0.21 × 10^9^/L) counts compared to the ISO group (*p* < 0.05). A similar trend was observed in the ISO + Dig group, indicating a comparable modulatory effect.

### 2.9. Cardiac Injury Marker (NT-proBNP)

**Figure 5:**
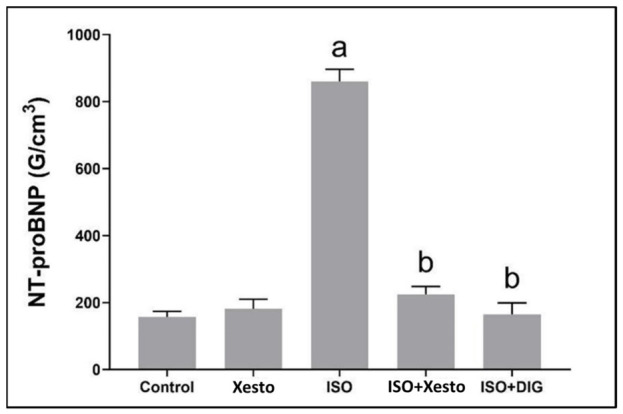
Serum NT-proBNP levels in each experimental group. Data are expressed as mean ± SEM (*n* = 7 per group). Statistical significance is indicated as: a *p* < 0.05 vs. Control; b *p* < 0.05 vs. ISO. Control: rats given normal saline. Xesto: rats given *Xestospongia testudinaria* extract (15 mg/kg/day). ISO: rats administered isoprenaline (10 mg/kg/day). ISO + Xesto: rats administered ISO followed by Xesto extract (15 mg/kg/day). ISO + DIG: rats administered ISO followed by digoxin (10 mg/kg/day).

Serum NT-proBNP levels were markedly elevated in the ISO-treated group (860.0 ± 36.74 G/cm^3^) compared to the control group (*p* < 0.05), confirming the development of cardiac injury (Figure 5). Treatment with Xesto or digoxin significantly reduced NT-proBNP levels compared to the ISO group (*p* < 0.05). There was no significant difference between the Xesto-only and control groups, suggesting that Xesto did not exert adverse effects on cardiac function under normal conditions.

### 2.10. Oxidative Stress and Antioxidant Parameters

The activities of serum antioxidant enzymes and the level of lipid peroxidation are presented in Table 6. ISO administration markedly reduced the activities of superoxide dismutase (SOD) and catalase (CAT), accompanied by a significant elevation in malondialdehyde (MDA) levels compared to the control group (*p* < 0.05), indicating oxidative stress-induced myocardial damage.

Treatment with Xesto or digoxin significantly restored SOD and CAT activities while reducing MDA levels compared to the ISO group (*p* < 0.05). The antioxidant activities in the Xesto-treated group were comparable to those of the digoxin-treated rats, suggesting that Xesto exerts potent free radical–scavenging and cardioprotective effects through oxidative stress attenuation (Table 7).

**Figure 6:**
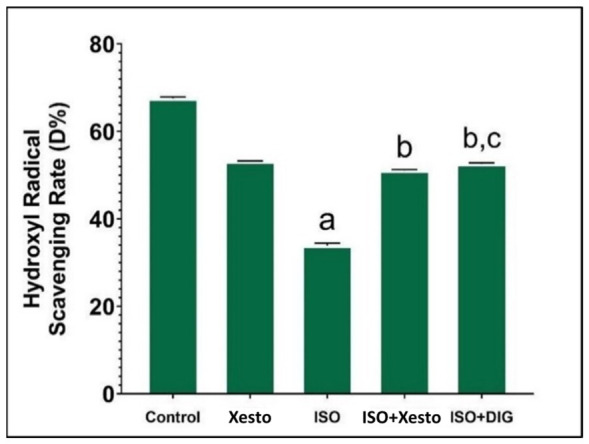
Hydroxyl radical scavenging activity in the serum of rats in each experimental group. Data are expressed as mean ± SEM (*n* = 7 per group). Statistical significance is indicated as: a *p* < 0.05 vs. Control; b *p* < 0.05 vs. ISO; c *p* < 0.05 vs. ISO + Xesto. Control: rats given normal saline. Xesto: rats given *Xestospongia testudinaria* extract (15 mg/kg/day). ISO: rats administered isoprenaline (10 mg/kg/day). ISO + Xesto: rats administered ISO followed by Xesto extract (15 mg/kg/day). ISO + DIG: rats administered ISO followed by digoxin (10 mg/kg/day).

The hydroxyl radical scavenging activity of serum samples from each experimental group is presented in Figure 6. The ISO-treated group showed a marked reduction in scavenging activity (*p* < 0.05) compared to the control, indicating enhanced oxidative stress and radical generation during cardiac injury.

Treatment with Xesto significantly improved hydroxyl radical scavenging activity relative to the ISO group (*p* < 0.05). The combination of ISO + Xesto demonstrated a scavenging rate comparable to the digoxin-treated group, suggesting that Xesto supplementation effectively mitigated free radical-induced oxidative damage and contributed to improved redox balance in the heart failure model.

### 2.11. Histopathological Analysis Findings

**Figure 7:**
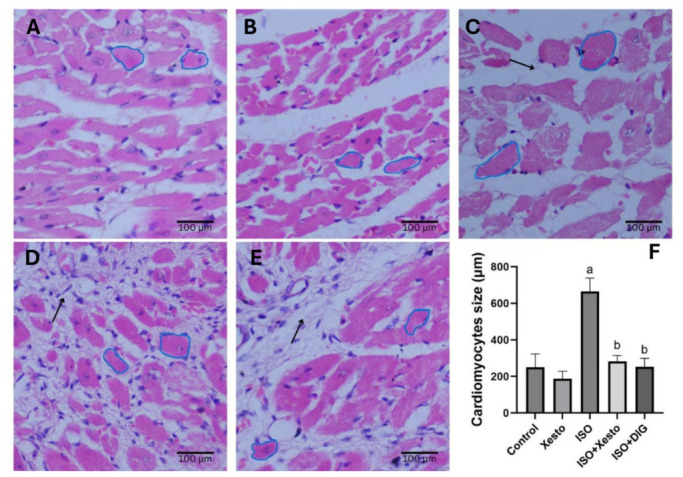
Representative H&E-stained cardiac tissue sections from the experimental groups observed at 400× magnification: (**A**) Control, (**B**) Xesto-only, (**C**) ISO, (**D**) ISO + Xesto, and (**E**) ISO + Digoxin, (**F**) Quantitative analysis of cardiomyocyte size. Blue outlines indicate cardiomyocyte boundaries used for morphometric analysis, while black arrows indicate histopathological alterations associated with ISO-induced myocardial injury. Cardiomyocyte size was quantified using ImageJ software. Data are expressed as mean ± SEM (*n* = 7 per group). Statistical significance is indicated as: a *p* < 0.05 vs. Control; b *p* < 0.05 vs. ISO. Scale bar = 100 μm. Control: rats given normal saline. Xesto: rats given *Xestospongia testudinaria* extract (15 mg/kg/day). ISO: rats administered isoprenaline (10 mg/kg/day). ISO + Xesto: rats administered ISO followed by *Xestospongia testudinaria* extract (15 mg/kg/day). ISO + DIG: rats administered ISO followed by digoxin (10 mg/kg/day).

Representative H&E-stained heart sections from each experimental group are shown in Figure 7. Cardiomyocyte size was quantified using ImageJ software (version 1.53e). The ISO-treated group exhibited marked histopathological alterations, including myocardial fiber disorganization and inflammatory cell infiltration.

**Figure 8:**
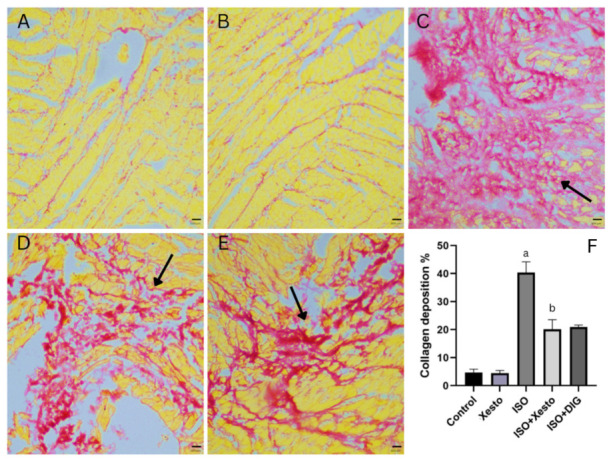
Representative Picrosirius Red-stained cardiac tissue sections from the experimental groups observed at 100× magnification: (**A**) Control, (**B**) Xesto-only, (**C**) ISO, (**D**) ISO + Xesto, and (**E**) ISO + Digoxin, (**F**) quantitative analysis of collagen deposition expressed as percentage collagen area. Black arrows indicate areas of collagen deposition and myocardial fibrosis. Collagen deposition was quantified using ImageJ software and expressed as percentage collagen area. Data are expressed as mean ± SEM (*n* = 7 per group). Statistical significance is indicated as: a *p* < 0.05 vs. Control; b *p* < 0.05 vs. ISO. Scale bar = 100 μm. Control: rats given normal saline. Xesto: rats given *Xestospongia testudinaria* extract (15 mg/kg/day). ISO: rats administered isoprenaline (10 mg/kg/day). ISO + Xesto: rats administered ISO followed by *Xestospongia testudinaria* extract (15 mg/kg/day). ISO + DIG: rats administered ISO followed by digoxin (10 mg/kg/day).

Quantitative analysis revealed a significant increase (*p* < 0.05) in cardiomyocyte size in the ISO group compared to the control (Figure 8). Treatment with Xesto or digoxin significantly reduced cardiomyocyte size relative to the ISO group (*p* < 0.05), indicating attenuation of ISO-induced cardiac hypertrophy.

## 3. Discussion

The present study provides compelling evidence that Xesto confers significant cardioprotective, antioxidant, and antihypertrophic effects in ISO-induced heart failure rats. These protective effects were demonstrated through improvements in haemodynamic, biochemical, and histopathological parameters, comparable to those observed with the reference drug digoxin.

Phytochemical characterization is essential for identifying bioactive constituents responsible for therapeutic activity [21]. In this study, Xesto contained phenolics, tannins, saponins, triterpenoids, and steroids, while alkaloids, flavonoids, and cardiac glycosides were absent. The notably high total phenolic content and measurable flavonoid content indicate substantial redox capacity, suggesting strong potential to neutralize reactive oxygen species. Phenolic compounds are well established for their antioxidative properties and ability to attenuate oxidative stress [22], while triterpenoids have been associated with anti-inflammatory and cytoprotective effects [23]. Steroidal and terpenoid metabolites isolated from marine organisms have also been reported to exhibit antioxidant, anti-inflammatory, and cytoprotective activities through modulation of oxidative stress pathways and preservation of cellular integrity [24,25]. These biological properties may contribute to the restoration of endogenous antioxidant defenses and attenuation of cardiac injury observed following Xesto treatment. Similar phytochemical richness has been reported in other Xestospongia species, including *Xestospongia testudinaria*, which produces a diverse range of secondary metabolites such as sterols, sterol esters, brominated fatty acids, polyacetylenes, and terpenoid-related compounds [17,26,27]. Previous studies have identified compounds including xestosterol derivatives, langcosterol A, and testusterol, many of which exhibit antioxidant, cytoprotective, antimicrobial, and cytotoxic activities [28,29,30].

Although targeted metabolomic profiling was not performed in the present study, these previously reported metabolites may provide a plausible biochemical basis for the antioxidant and cardioprotective effects observed. Nevertheless, the contribution of individual compounds remains speculative and requires further confirmation through comprehensive chemical characterization and bioactivity-guided isolation studies. Thus, the phytochemical composition of Xesto provides a rational biochemical basis for the cardioprotective effects observed in this model.

Physiological indicators such as body weight, heart weight, and blood pressure reflect systemic stress and myocardial remodeling [31,32,33]. ISO administration resulted in increased heart weight and altered body weight, consistent with sympathetic overstimulation and hypertrophic remodeling [34,35,36]. Treatment with Xesto significantly attenuated these changes, indicating suppression of maladaptive hypertrophy and preservation of myocardial mass. ISO-induced elevations in systolic and diastolic blood pressure were also markedly reduced following Xesto treatment. Phenolic-rich compounds have been shown to improve endothelial function and reduce oxidative vascular injury [20,37,38], which may partly explain the haemodynamic stabilization observed.

ISO exposure also produced significant haematological disturbances, including reduced haemoglobin levels and elevated leukocyte and neutrophil counts. Oxidative stress is known to contribute to inflammatory activation and hematologic imbalance [39], and catecholamine-induced injury models demonstrate systemic inflammatory responses [40,41]. Restoration of these parameters following Xesto treatment suggests attenuation of inflammatory stress and improved systemic homeostasis.

Biochemical analysis revealed elevated AST, ALT, and urea levels in ISO-treated rats, indicating hepatic and renal stress. Similar hepatorenal complications have been reported in ISO-induced cardiotoxicity [42]. Phenolic-rich marine extracts have demonstrated protective effects against oxidative injury and mitochondrial dysfunction [43,44], consistent with the reductions observed in Xesto-treated animals. These findings indicate that Xesto exerts multi-organ protective effects in addition to cardiac preservation.

NT-proBNP serves as a sensitive biomarker of ventricular stress and heart failure progression [45,46,47]. SO administration significantly elevated NT-proBNP levels, confirming myocardial strain, consistent with β-adrenergic overstimulation models [48,49]. Treatment with Xesto significantly reduced NT-proBNP concentrations. Antioxidant-based therapeutic strategies have been shown to mitigate cardiac overload and oxidative myocardial injury [50,51], supporting the observed cardioprotective profile of Xesto.

Oxidative stress is a central driver of ISO-induced cardiac injury, characterized by excessive reactive oxygen species generation and depletion of endogenous antioxidant enzymes [52,53]. In the present study, ISO significantly decreased SOD and CAT activities while increasing MDA levels, in agreement with previous reports [54]. Xesto treatment restored antioxidant enzyme activities and reduced lipid peroxidation. Enhancement of endogenous antioxidant systems alongside suppression of membrane lipid oxidation indicates effective redox stabilization.

To explore a potential molecular mechanism underlying this antioxidant effect, molecular docking was performed targeting KEAP1, a cytoplasmic regulator of NRF2. Under basal conditions, KEAP1 promotes NRF2 degradation, thereby limiting transcription of antioxidant genes [55,56]. Disruption of the KEAP1–NRF2 interaction permits NRF2 nuclear translocation and activation of cytoprotective enzymes such as SOD and CAT [57,58]. In this study, trans-phytol demonstrated favorable binding within the Kelch domain of KEAP1, interacting with ARG415 and adjacent residues involved in NRF2 recognition. Although computational in nature, this interaction suggests possible interference with the KEAP1–NRF2 complex formation, which may contribute to the observed restoration of endogenous antioxidant enzyme activity and reduction in oxidative stress in vivo. Nevertheless, the docking results should be interpreted as a preliminary mechanistic insight rather than direct evidence of KEAP1–NRF2 pathway activation, as no experimental assessment of KEAP1 or NRF2 expression was performed in the present study.

Furthermore, the present findings are insufficient to establish trans-phytol as the principal bioactive constituent responsible for the observed cardioprotective effects. Given the complexity of the crude methanolic extract and the presence of multiple phytochemical classes, the observed biological activity may arise from the combined or synergistic actions of several constituents. Therefore, future studies involving bioactivity-guided fractionation, compound isolation, and pathway-specific validation are warranted to identify the key active compounds and further elucidate their mechanisms of action. Despite these limitations, the docking findings complement the biochemical and antioxidant data and provide a plausible mechanistic basis for the observed biological effects.

Histopathological evaluation confirmed that ISO induced myocardial fiber disorganization, inflammatory infiltration, and collagen deposition, consistent with classical descriptions of ISO-induced myocardial necrosis and remodeling [59]. Xesto treatment preserved myocardial architecture and significantly reduced collagen accumulation. Marine-derived bioactive compounds have demonstrated antifibrotic properties through suppression of extracellular matrix deposition and modulation of remodeling pathways [60,61]. The reduction in collagen deposition observed here suggests that Xesto mitigates structural remodeling through coordinated antioxidant and antifibrotic mechanisms.

Overall, the integration of haemodynamic stabilization, normalization of biochemical injury markers, restoration of endogenous antioxidant defenses, attenuation of ventricular stress, and preservation of myocardial structure indicates that Xesto exerts multi-target cardioprotection. These findings align with emerging evidence supporting marine-derived natural products as modulators of oxidative and inflammatory cascades in cardiovascular diseases [62,63,64].

### Limitation

A limitation of the present study is the absence of comprehensive chemical characterization and quantitative profiling of the methanolic extract. Although preliminary phytochemical screening, TPC, TFC, and molecular docking analyses were performed, the specific compounds responsible for the observed cardioprotective effects could not be experimentally identified. Furthermore, the observed biological effects may result from the synergistic actions of multiple phytochemical constituents rather than a single bioactive compound. Therefore, direct structure–activity relationships could not be established. Future studies employing GC-MS, LC-MS/MS, metabolomic profiling, and bioactivity-guided fractionation are warranted to identify and quantify the principal bioactive constituents and elucidate their molecular mechanisms of action.

In addition, only a single dose of Xesto (15 mg/kg) was evaluated in the present study. This dose was selected based on preliminary studies that identified it as the most effective dose for subsequent efficacy evaluation. Nevertheless, the use of a single dose precluded assessment of dose–response relationships and determination of the therapeutic range. Future studies employing multiple dose levels are warranted to establish dose-dependent efficacy and safety profiles.

Furthermore, the present findings were obtained using an isoprenaline-induced rat model, which may not fully replicate the complexity of human cardiovascular disease. Although the observed cardioprotective and antioxidant effects suggest potential therapeutic relevance, differences in physiology, metabolism, and disease progression between animal models and humans should be considered when interpreting the findings. Therefore, further preclinical and clinical studies are required to confirm the translational potential of Xesto.

## 4. Materials and Methods

### 4.1. Molecular Docking

The crystal structure of human KEAP1 (PDB ID: 4L7B) was retrieved from the Protein Data Bank. The three-dimensional structures of trans-phytol, nonanedioic acid, and digoxin (reference ligand) were obtained from the PubChem database.

Protein and ligand preparation were performed using UCSF ChimeraX (v1.12). Co-crystallized ligands and water molecules were removed, and polar hydrogens were added. Protonation states were adjusted to physiological pH (7.4), and structures were optimized prior to docking.

Molecular docking simulations were conducted using AutoDock Vina (version 1.2.0) implemented through the CB-Dock2 platform. The binding cavity was defined based on the location of the native ligand within the Kelch domain of KEAP1. Docking was performed in triplicate for each ligand, and the conformation with the lowest binding energy (kcal/mol) was selected for further analysis [65].

Protein–ligand interactions, including hydrogen bonding, van der Waals forces, and hydrophobic interactions, were analyzed using BIOVIA Discovery Studio Visualizer (v25.1.0.24284).

### 4.2. Animals

Thirty-five healthy male Wistar rats weighing 200–250 g were used in this study. The animals were not genetically modified and had not undergone any previous experimental procedures prior to study initiation. All animals were acclimatized for one week under standard laboratory conditions and maintained at a temperature of 22 ± 2 °C with a 12 h light/dark cycle. Standard rodent chow and tap water were provided ad libitum throughout the study period. To minimize potential confounding factors, all animals were housed under identical environmental conditions, including temperature, light–dark cycle, diet, and access to water. Treatments and measurements were conducted according to the same experimental schedule for all groups throughout the study period. All animal experiments were conducted in accordance with the guidelines and regulations of the Management & Science University Animal Ethics Committee (MSUAEC). The study protocol was reviewed and approved under approval number EA-L3-01-FHLS-2023-10-0002 on 20 December 2023. All methods were carried out in compliance with the ARRIVE guidelines and institutional regulations for the care and use of laboratory animals. All healthy male Wistar rats that met the specified age and weight requirements were included in the study. Animals were excluded only if they developed severe illness, sustained injury unrelated to the experimental procedures, or died before completion of the study. No animals met these exclusion criteria, and all animals completed the experimental protocol and were included in the final analyses.

### 4.3. Sample Size Calculation

The sample size was determined using the resource equation method as described by Charan and Kantharia (2013) [66]. An initial sample size of six animals per group was estimated to be sufficient for detecting biologically meaningful differences among the experimental groups. To account for a potential attrition rate of approximately 15% during the induction and treatment phases, the sample size was increased to seven animals per group. Therefore, a total of 35 male Wistar rats were included in the study.

### 4.4. Collection and Preparation of Xestospongia testudinaria Methanolic Extract

The sponge was collected by divers from the coastal water of Sabah, Malaysia after appropriate permission, and immediately frozen following the collection. The Red Sea Marine Sponge *Xestospongia testudinaria* exhibits a distinctive volcano-like morphology characterized by prominent longitudinal ridges on its pink or pale red-brown exterior. Its skeletal structure comprises an irregular isotropic or alveolar reticulation of spicule bundles, typically 2–6 spicules in cross-section, with individual spicules showing variability in length between 100 and 400 μm. The sponge was identified as *Xestospongia testudinaria* by a marine biologist at the Borneo Marine Research Institute, Universiti Malaysia Sabah in Malaysia.

The specimens were thawed at room temperature for approximately six hours. The weight measurements were recorded at hourly intervals to assess the diminution of water content. Upon reaching a weight stabilization phase (after 5–6 h), the samples were then dried in a hot air oven at 60 °C for 48 h. Subsequently, the desiccated sponge material was then weighed and pulverized into a fine powder. For sample preparation, 1500 g of the dried sample were macerated in 6000 mL of methanol for a period of 72 h at room temperature. The resultant methanolic extract was then filtered with Whatman’s filter paper No. 1 and concentrated under reduced pressure using a rotary evaporator with a maximum temperature setting of 40 °C. The sample was then freeze-dried to yield a dry powdered extract and stored in a freezer at 4 °C until further analysis.

Methanol was selected as the extraction solvent because of its ability to efficiently extract a broad spectrum of polar and moderately non-polar secondary metabolites, including phenolic compounds, terpenoids, and steroids commonly reported in marine sponge species. Previous studies have also shown that organic extracts of *Xestospongia testudinaria* exhibit greater biological activity than aqueous extracts, suggesting that bioactive constituents are more effectively recovered using organic solvents [67]. Drying at 60 °C was performed to remove residual moisture while minimizing excessive thermal degradation of sponge-derived metabolites. Maceration for 72 h at room temperature was employed to facilitate solvent penetration and metabolite extraction while reducing the risk of thermal decomposition of heat-sensitive compounds. Concentration under reduced pressure at 40 °C followed by freeze-drying was used to preserve the chemical integrity and stability of the extracted metabolites prior to storage and subsequent analyses.

### 4.5. Experimental Groups

After a 2-week acclimatization period, each animal was assigned a unique identification number and allocated to one of the five experimental groups. Thirty-five Wistar rats were divided into five groups (*n* = 7 per group) as follows:

- Group I (Control): Received normal saline throughout the 28-day study period.
- Group II (Xesto-only): Received *Xestospongia testudinaria* methanolic extract (Xesto; 15 mg/kg/day, oral gavage) for 28 days.
- Group III (ISO-only): Received isoprenaline (ISO; 10 mg/kg/day, subcutaneous injection) for 14 consecutive days and normal saline for the following 14 days [68].
- Group IV (ISO + Xesto): Received ISO (10 mg/kg/day, subcutaneous) for 14 days followed by Xesto (15 mg/kg/day, oral gavage) for 14 days [69].
- Group V (ISO + Digoxin): Received ISO (10 mg/kg/day, subcutaneous) for 14 days followed by digoxin (10 mg/kg/day, oral gavage) for 14 days.

ISO, Xesto extract, and digoxin were freshly prepared in normal saline prior to administration. Heart failure was induced during the 14-day induction phase in Groups III–V through daily ISO injections, while Groups I and II received equivalent volumes of normal saline. This induction period was followed by a 14-day treatment phase, during which the Xesto-only and ISO + Xesto groups received Xesto (15 mg/kg/day, oral gavage) and the ISO + Digoxin group received digoxin (10 mg/kg/day, oral gavage). The Control and ISO-only groups continued to receive normal saline by oral gavage throughout the treatment period.

Investigators were aware of group allocation during animal allocation and treatment administration due to the nature of the experimental procedures. However, outcome assessment of histopathological images was performed in a blinded manner during image analysis.

At the end of the experiment (Day 29), the rats were anaesthetized before being sacrificed to ensure they were unconscious and did not experience pain. Euthanasia was then performed following the American Veterinary Medical Association Guidelines for the Euthanasia of Animals. This approach was selected to minimize suffering and ensure humane treatment in accordance with ethical standards.

### 4.6. Phytochemical Analysis of Xestospongia testudinaria Methanolic Extract

Phytochemical screening of Xesto for the presence of alkaloids, flavonoids, phenolics, cardiac glycosides, saponins, triterpenoids, and steroids was carried out.

#### 4.6.1. Test for Alkaloids

The extract was stirred with 1% HCl and the mixture was warmed and filtered [70]. A total of 2 mL of filtrate was treated separately with (a) with few drops of potassium mercuric iodide (Mayer’s reagent) and (b) potassium bismuth iodide (Dragendorff’s reagent). The formation of a cream-coloured precipitate in Mayer’s test and an orange-red precipitate in Dragendorff’s test indicated the presence of alkaloids.

#### 4.6.2. Test for Flavonoids

To qualitatively identify flavonoids, two assays were conducted. In the alkaline reagent test, 2–3 drops of sodium hydroxide solution were introduced into 2 mL of the extract, producing an intense yellow colour that faded upon the addition of dilute hydrochloric acid (HCl), confirming the presence of flavonoids. In the Shinoda test, 10 drops of dilute HCl and a small piece of magnesium ribbon were added to 1 mL of the extract, and the development of a pink to red coloration provided additional evidence of flavonoids [71].

#### 4.6.3. Test for Phenolics and Tannins

For the qualitative detection of phenolic compounds and tannins, two tests were conducted. In the Ferric Chloride Test, 2 mL of 5% neutral ferric chloride solution was added to 1 mL of the extract, and the appearance of red, green, purple, or blue coloration indicated the presence of phenolic compounds and tannins [72]. In the Lead Acetate Test, 1 mL of lead tetraacetate solution was added to 0.5 mL of the extract, where the formation of a precipitate confirmed the presence of phenolic and tannin constituents [73].

#### 4.6.4. Cardiac Glycosides Determination

Five millilitres of the extract solution (10 mg/mL in methanol) were mixed with 2 mL of glacial acetic acid containing a drop of ferric chloride (FeCl_3_) solution. Subsequently, 1 mL of concentrated sulfuric acid (H_2_SO_4_) was carefully added to form a distinct layer. The appearance of a brown ring at the interface indicated the presence of deoxysugars, characteristic of cardiac glycosides [74].

#### 4.6.5. Test for Saponins

Five millilitres of the extract were mixed with a drop of sodium carbonate (Na_2_CO_3_) solution and shaken vigorously. The formation of a stable froth accompanied by a white precipitate after five minutes confirmed the presence of saponins [75].

#### 4.6.6. Test for Triterpenoids

One millilitre of the extract was treated with 2 mL of trichloroacetic acid, and the appearance of a red precipitate in the mixture indicated the presence of terpenoids [71].

#### 4.6.7. Test for Steroids

Two millilitres of the extract were mixed with an equal volume of chloroform in a test tube, followed by the careful addition of 1 millilitre of concentrated sulfuric acid. The appearance of a reddish-brown colouration indicated the presence of steroids [21,76].

#### 4.6.8. Total Phenolic Content

The total phenolic content (TPC) was determined by the spectrophotometric method [77]. Gallic acid was used as the standard in the Folin–Ciocalteu technique to calculate the total phenolic content [78]. Briefly, 400 μg of the crude extract was mixed with 200 μL of Folin–Ciocalteu reagent and 3.16 mL of distilled water. After standing for 30 to 8 min, 600 μL of 20% sodium carbonate solution was added. The mixture was then incubated at 20 °C for two hours, and its absorbance was measured at 636 nm using a DR6000 UV–Vis spectrophotometer (Hach Company, Loveland, CO, USA). The TPC was calculated from a gallic acid calibration curve ranging from 0 to 160 μg/mL (R^2^ = 0.9901) and expressed as milligrams of gallic acid equivalents (GAE) per gram of extract. All measurements were performed in triplicate.

#### 4.6.9. Total Flavonoid Content

The total flavonoid content (TFC) was determined according to the method described by Park et al. (2008) [79]. In a 10 mL test tube, 0.3 mL of the extract was combined with 3.4 mL of 30% methanol, 0.15 mL of sodium nitrite (NaNO_2_, 0.5 M), and 0.15 mL of aluminium chloride hexahydrate (AlCl_3_·6H_2_O, 0.3 M). After a 5 min incubation, 1 mL of sodium hydroxide (NaOH, 1 M) was added, and the mixture was thoroughly mixed. The absorbance was then measured at 506 nm against a reagent blank. A standard calibration curve was prepared using rutin solutions ranging from 0 to 100 mg/L, following the same procedure. The total flavonoid content was expressed as milligrams of rutin equivalents (RE) per gram of dried extract.

### 4.7. Body and Heart Weight Analysis

Body weight (g) was measured in the Wistar rats at baseline, post-ISO induction, and post-treatment in all the groups. Measurements were obtained in the morning using a calibrated digital balance.

At the end of the experimental period, all rats were anaesthetized and euthanized. Hearts were carefully excised, rinsed with ice-cold saline to remove residual blood, and blotted dry with filter paper. Each heart was immediately weighed using a calibrated analytical balance, and the absolute heart weight (g) was recorded for each animal.

### 4.8. Systolic and Diastolic Blood Pressure Measurement

The blood pressure was measured weekly during the treatment period using the non-invasive tail-cuff method with the CODA™ blood pressure monitoring system (Kent Scientific, Torrington, CT, USA). Each rat was placed individually in a restrainer, with its tail positioned inside the cuff, and acclimated on a warming pad for 10–15 min. The CODA system recorded the blood pressure during sequential cuff inflation and deflation for 15 cycles per session. Six consecutive readings were obtained. The highest and lowest values were excluded and the remaining measurements were averaged for analysis.

### 4.9. Biochemical and Hematological Analysis

Blood samples were collected from the rats via retro-orbital puncture using plain tubes. The samples were allowed to clot at room temperature and then centrifuged at 3000 rpm for 10 min to separate the serum. The resulting serum was collected and stored at −20 °C until further analysis.

Serum biochemical parameters, including aspartate aminotransferase (AST), alanine aminotransferase (ALT), and urea, were quantified using commercially available diagnostic kits in accordance with the manufacturer’s protocols. Absorbance was measured using a DR6000 UV–Visible spectrophotometer (Hach Company, Loveland, CO, USA).

For hematological analysis, the blood was collected using heparinized capillary tubes. Approximately 1 mL of blood was transferred into EDTA-coated tubes for hematological analysis. Hematological parameters, including haemoglobin concentration, total leukocyte count, and differential leukocyte count (neutrophils, lymphocytes, and monocytes) were measured using an automated hematology analyzer (Beckman Coulter, Inc., Brea, CA, USA).

### 4.10. Analysis of Cardiac Biomarker (NT-proBNP)

Serum levels of N-terminal pro-B-type natriuretic peptide (NT-proBNP) were quantified using a commercially available enzyme-linked immunosorbent assay (ELISA) kit (Elabscience Biotechnology Inc., Wuhan, China), following the manufacturer’s instructions. All reagents and samples were brought to room temperature before analysis. The optical density (OD) was measured at 450 nm using a microplate reader (Bio-Rad, Hercules, CA, USA), and NT-proBNP concentrations were calculated from a standard calibration curve.

### 4.11. Oxidative Stress and Antioxidant Biomarker Assays

Oxidative stress markers and antioxidant enzyme activities were evaluated using commercially available assay kits (Solarbio, Beijing, China) in accordance with the manufacturer’s instructions [80,81].

Superoxide dismutase (SOD) activity was determined using the SOD Activity Assay Kit (Elabscience Biotechnology Inc., Wuhan, China), with absorbance recorded at 560 nm using a microplate reader (Bio-Rad, Hercules, CA, USA). Catalase (CAT) activity was measured using the CAT Activity Assay Kit (Elabscience Biotechnology Inc., Wuhan, China), and absorbance was read at 240 nm. Lipid peroxidation was assessed by quantifying malondialdehyde (MDA) levels with the MDA Assay Kit (Elabscience Biotechnology Inc., Wuhan, China); absorbance was recorded at 450, 532, and 600 nm. Hydroxyl radical scavenging activity was determined using the Hydroxyl Radical Scavenging Assay Kit (Elabscience Biotechnology Inc., Wuhan, China), with absorbance measured at 546 nm. All calculations were performed according to the formulas provided in the respective assay manuals.

### 4.12. Histopathological Analysis

Excised heart tissues were fixed in 10% neutral-buffered formalin for 24 h and subsequently processed through a standard histological procedure comprising dehydration, clearing, and paraffin embedding. The paraffin-embedded tissues were sectioned at 5 µm thickness using a rotary microtome and mounted onto glass slides. For general morphological assessment, the sections were stained with haematoxylin and eosin (H&E). Additional sections were stained with Picrosirius Red to evaluate collagen deposition and identify fibrotic regions. Stained sections were examined under a light microscope, and quantitative analysis of cardiomyocyte dimensions and collagen area percentage was performed using ImageJ software (NIH, Bethesda, MD, USA).

### 4.13. Statistical Analysis

Data normality was verified via the Shapiro–Wilk test. All data were expressed as mean ± standard error of the mean (SEM). Statistical comparisons among groups were performed using one-way analysis of variance (ANOVA), followed by Tukey’s post hoc test for multiple comparisons. Analyses were conducted using GraphPad Prism software (version 10; GraphPad Software, Boston, MA, USA). A *p*-value < 0.05 was considered statistically significant.

## 5. Conclusions

In conclusion, Xesto exerts significant cardioprotective, antioxidant, and antihypertrophic effects in ISO-induced cardiac injury. The extract improved haemodynamic parameters, restored endogenous antioxidant enzyme activity, reduced NT-proBNP levels, and preserved myocardial architecture, with efficacy comparable to digoxin. The integration of biochemical, histological, and molecular docking findings suggests that modulation of oxidative stress pathways may contribute to the observed protective effects. Molecular docking analysis identified trans-phytol as a potential KEAP1-interacting compound; however, experimental validation is required to confirm its role in mediating the observed biological effects and to verify the involvement of KEAP1–NRF2 signaling.

As the present study utilized a crude methanolic extract, the specific compounds responsible for the observed cardioprotective effects could not be experimentally identified. Further studies involving comprehensive chemical characterization, metabolomic profiling, bioactivity-guided fractionation, and pathway-specific investigations are warranted to identify the principal bioactive constituents and elucidate their mechanisms of action. These findings support the potential of Xesto as a marine-derived therapeutic candidate for heart failure management.

## Figures and Tables

**Table 1:** Binding affinities and key interacting residues of Xesto-derived compounds and digoxin with KEAP1.

Ligand	Binding Energy (kcal/mol)	Hydrogen Bond Residues
Digoxin	−10.8	ARG415, TYR525
Trans-phytol	−6.3	ARG415
Nonanedioic acid	−4.3	GLU493

**Table 2:** Presence (+) or absence (−) of phytochemicals in the methanolic extract of *Xestospongia testudinaria*.

Phytochemicals	Inference
Alkaloids	−
Flavonoids	−
Phenolics and tannins	+
Cardiac Glycosides	−
Saponins	+
Triterpenoids	+
Steroids	+

**Table 5:** Serum AST, ALT, and urea levels in control and isoprenaline (ISO)-induced rats treated with *Xestospongia testudinaria* methanolic extract (Xesto) or digoxin.

Group	AST (U/L)	ALT (U/L)	Urea (mmol/L)
Control	87.57 ± 1.19	49.93 ± 1.48	5.94 ± 0.16
Xesto	79.14 ± 0.77	44.00 ± 1.60	4.33 ± 0.19
ISO	115.4 ± 2.96 ^a^	89.43 ± 1.56 ^a^	7.89 ± 0.22 ^a^
ISO + Xesto	105.7 ± 2.58 ^b^	74.29 ± 1.60 ^b^	7.10 ± 0.11 ^b^
ISO + DIG	96.29 ± 1.57 ^b,c^	60.57 ± 1.29 ^b,c^	6.26 ± 0.21 ^b,c^

Data are expressed as mean ± SEM (*n* = 7 per group). ^a^: *p* < 0.05 vs. CONTROL; ^b^: *p* < 0.05 vs. ISO; ^c^: *p* < 0.05 vs. ISO + Xesto. Xesto: *Xestospongia testudinaria* extract; ISO: isoprenaline; DIG: digoxin.

**Table 6:** Effect of isoprenaline on haemoglobin concentration, leukocyte count, and neutrophil count.

Group	Hemoglobin (g/dL)	Leukocyte (×10^9^ L)	Neutrophil (×10^9^ L)
Control	16.13 ± 0.50	9.17 ± 0.29	2.07 ± 0.13
Xesto	13.70 ± 0.27	5.02 ± 0.41	1.68 ± 0.23
ISO	11.88 ± 0.24 ^a^	15.42 ± 0.44 ^a^	8.20 ± 0.15 ^a^
ISO + Xesto	13.48 ± 0.19 ^b^	9.90 ± 0.30 ^b^	6.12 ± 0.21 ^b^
ISO + DIG	15.10 ± 0.24 ^b,c^	7.08 ± 0.26 ^b,c^	5.06 ± 0.09 ^b,c^

Data are expressed as mean ± SEM (*n* = 7 per group). ^a^: *p* < 0.05 vs. CONTROL; ^b^: *p* < 0.05 vs. ISO; ^c^: *p* < 0.05 vs. ISO + Xesto. Xesto: *Xestospongia testudinaria* extract; ISO: isoprenaline; DIG: digoxin.

**Table 7:** Effect of treatments on enzymatic antioxidant activities (SOD, CAT) and lipid peroxidation (MDA) in the serum of isoprenaline-induced heart failure rats.

Group	SOD (U/mL)	CAT (U/mL)	MDA (nmol/mL)
Control	13.33 + 0.03	14.23 + 0.43	0.71 + 0.01
Xesto	14.70 + 0.12	17.57 + 0.26	0.55 + 0.02
ISO	7.66 + 0.09 ^a^	9.73 + 0.34 ^a^	1.61 + 0.04 ^a^
ISO + Xesto	10.90 + 0.07 ^b^	12.09 + 0.19 ^b^	0.57 + 0.02 ^b^
ISO + DIG	12.40 + 0.04 ^b,c^	13.68 + 0.49 ^b,c^	0.68 + 0.03 ^b,c^

Data are expressed as mean ± SEM (*n* = 7 per group). Statistical significance: ^a^ *p* < 0.05 vs. control; ^b^ *p* < 0.05 vs. ISO; ^c^ *p* < 0.05 vs. ISO + Xesto. Xesto: *Xestospongia testudinaria* methanolic extract; ISO: isoprenaline; DIG: digoxin.

## Data Availability

All the data generated and analyzed during this study are included in this article.

## Abbreviations

The following abbreviations are used in this manuscript:

ALT	Alanine aminotransferase
ANOVA	Analysis of variance
AST	Aspartate aminotransferase
CAT	Catalase
CVD	Cardiovascular disease
DIG	Digoxin
EDTA	Ethylenediaminetetraacetic acid
ELISA	Enzyme-linked immunosorbent assay
H&E	Hematoxylin and eosin
HF	Heart failure
ISO	Isoprenaline
MDA	Malondialdehyde
MMP	Matrix metalloproteinase
NT-proBNP	N-terminal pro-B-type natriuretic peptide
ROS	Reactive oxygen species
SEM	Standard error of the mean
SOD	Superoxide dismutase
TFC	Total flavonoid content
TGF-β	Transforming growth factor-beta
TPC	Total phenolic content
WBC	White blood cell
Xesto	*Xestospongia testudinaria* methanolic extract
